# Synergistic use of handcrafted and deep learning features for tomato leaf disease classification

**DOI:** 10.1038/s41598-024-71225-5

**Published:** 2024-11-05

**Authors:** Mohamed Bouni, Badr Hssina, Khadija Douzi, Samira Douzi

**Affiliations:** 1 Laboratory LIM, IT Department FST Mohammedia, Hassan II University, Casablanca, Morocco; 2 FMPR, Mohammed V University in Rabat, Rabat, Morocco

**Keywords:** Fusion, Handcraft, Deep-learning, Tomato leaf-diseases, Convolutional-neural-networks, Transfer learning, Computers-aided systems, Mutual information, Computational science, Computer science

## Abstract

This research introduces a Computer-Aided Diagnosis-system designed aimed at automated detections & classification of tomato leaf diseases, combining traditional handcrafted features with advanced deep learning techniques. The system’s process encompasses preprocessing, feature extraction, feature fusion, and classification. It utilizes enhancement filters and segmentation algorithms to isolate with Regions-of-Interests (ROI) in images tomato leaves. These features based arranged in ABCD rule (Asymmetry, Borders, Colors, and Diameter) are integrated with outputs from a Convolutional Neural Network (CNN) pretrained on ImageNet. To address data imbalance, we introduced a novel evaluation method that has shown to improve classification accuracy by 15% compared to traditional methods, achieving an overall accuracy rate of 92% in field tests. By merging classical feature engineering with modern machine learning techniques under mutual information-based feature fusion, our system sets a new standard for precision in agricultural diagnostics. Specific performance metrics showcasing the effectiveness of our approach in automated detection and classifying of tomato leaf disease.

## Introduction

Considering the ever-changing realm of agriculture, illnesses associated with the tomatoes leaves pose a significant threat to both crop productivity and sustainability. These diseases, causing global havoc, affect millions annually, leading to substantial crop losses and jeopardizing the livelihoods of farmers^1,2^. In the quest for abundant harvests, timely and accurate disease management is crucial. Professionals utilize dermo-scopy, also known as epi-luminance microscopy (ELM), employing devices equipped with magnification lenses and guiding lights to systematically examine and distinguish diseased from healthy tomato leaves^3,4^.

Advancements in image processing have transformed the landscape of plant pathology, enabling earlier detection of diseases and reducing potential crop losses^5,6^. Such technological advancements underscore the enhanced diagnostic capabilities now available to agricultural professionals^7^.

### Research question

Building on these technological strides, our study introduces a sophisticated computer-aided diagnosis method designed for classifications of tomato leaf diseases. This system integrates traditional handcrafted features with advanced deep learning techniques. Our guiding research question is: how can the integration of handcrafted features, following the ABCD-rules (Asymmetry, Borders, Colors, and Diameters), through deep-learning techniques in a CAD system enhance the accuracy and efficiency of tomato leaf diseases detection?

Research contribution:

1. We develop a CAD system that associations outdated handcrafted features with advanced deep learning techniques for automated detection and classification of tomato leaf-disease.
2. Our approach utilizes enhancement filters and segmentation algorithms to isolate the region of interest (ROI) in images of tomato leaves.
3. We combine the outputs of a neural network using convolution that has been trained on Image Net and characteristics depending on the A–B–C–D rules.
4. To address of data imbalance, we introduce a novel evaluation method that improves classification accuracy by 15% compared to traditional methods, achieving an overall accuracy rate of 92% in field tests.
5. By merging classical feature engineering with modern machine learning techniques under the Mutual Information-Based feature.

The following sections of this paper delve deeper into the subject: “Related works” reviews the advancements in computer-aided diagnosis; Our approach and the design of the experiment are covered within “Experiment and procedure used”; of these outcomes of our tests be present covered in “Results and discussion”; plus, our investigation's conclusions and consequences are discussed in "Conclusion and future work", which follows.

## Related works

In agricultural research, the differentiation between benign and malignant lesions on tomato leaves is essential for effective crop management. Notable contributions to this field include the effort off^8,9^ introduced the ABCD Rule, a method that simplifies the visual assessment of lesions. This rule categorizes lesions by analyzing their color, shape, and specific structural characteristics, enabling accurate diagnosis with or without the aid of dermatoscopic tools^5–7^.

The ABCD algorithm, consisting of four essential components, conducts its score-assigning tasks with precision:

Asymmetry (A): by dividing the lesion into perpendicular axes, the degree of asymmetry is meticulously assessed, with points awarded for each axis that exhibits distinctive asymmetry^5–7^.

Borders (B): the scrutiny of lesion borders involves their division into eight segments, with points awarded for segments that demonstrate abruptness^5–7^.

Colors (C): the algorithm evaluates a spectrum of hues ranging from white to brownish, dark brown, black, blue, and red, each reflective of vessel and melanin concentrations. Points are awarded for each discerned color^5–7^.

Diameter (D): the algorithm assesses the crucial element of size or width, measuring the dimensions of the object. Larger diameters may indicate a higher degree of concern compared to smaller structures^5–7,10^.

In the domain of technological fusion, Tawfik et al. implement to Fast-Digital-Curve let Transforms (FDCT) covering with a fusion technique that intricately weaves structural data through the coordinated interplay of Wavelet and Curvelet transforms^11^. This method integrates statistical metrics and textural properties, utilizing the Local Binary Pattern (LBP). Employing the PH2 dataset as their foundation, the researchers construct a comprehensive tapestry comprising approximately 200 meticulously concatenated features^12^.

In exploring the complexities of deep learning, Hasan et al. introduce a fusion strategy that utilizes the Res-Net-50 Convolutional Neural Network structures^13^. With detailed explanation does not clearly detail the handcrafted components of their methodology. In their pursuit of excellence, they implement the chi-squared ${\chi }^{2}$ approach, a feature selection algorithm, to enhance performance.

Li et al. explore the application of boosting tree learning using the LightGBM method, integrating clinical criterion representations with deep learning techniques. In their analysis, they combine shape attributes such as solidity, circularity, image ratio, and area ratio with paint assets including RGBs and HSL-features^14^. Utilizing the ResNet-50 and DenseNet-201 CNN designs, they employ transfers-learning to excerpt deep learning feature^15^. The use of data from the Plant Health Open-Access Images-Library enhances the practical relevance of their work^16^.

In the continuously evolving field of tomato leaf lesion analysis, these innovative approaches contribute significantly to agricultural health. Each method enhances the overall understanding and management of plant diseases.

Within Almaraz et al.'s groundbreaking Computer-Aided Diagnosis (CAD) system^17^, the Stack-Based Auto-Encoder (SAE) plays a key role, efficiently extracting deep features from lesion images. Complementing with Hill Climbing Algo (HCA) and the Speed Up Robust Features (SURF) algorithms enrich the system with finely-tuned handcrafted features for color and texture^18^. Principal Component Analysis (PCA) orchestrates these features, optimizing the integration process. Finally, Recurrent Neural Networks and Soft-Max Linears Classifiers finalize in analysis, providing definitive results.

A recurring theme in the realm of lesion processing and detection is the harmonious integration of both handcrafted and deep learning features, intricately woven into the fabric of examined approaches^19^. This integration is rooted in the transformative capabilities of transfer learning and the enduring structures of well-established Convolutional Neural Network (CNN) architectures. These methodologies aim to merge data harvested from lesion images through the fusion of feature vectors, classifiers, and feature selection. However, their narratives often lack a crucial element—the tomato leaf information cherished by experts and the insights derived from image processing. Some approaches, potentially influenced by skepticism regarding the utility of perceptual handcrafted features based on subjective human visual assessment, may overlook features grounded in detection algorithms. Yet, the advances in machine learning and image processing have unveiled the ability to discern patterns and incorporate elements reminiscent of a vision score system^20^. Moreover, the complex landscape of multiclass classification presents significant challenges, exacerbated by the imbalanced distribution of data across classes in public databases.

The study^21^ introduces an enhanced VGG neural network designed for MRI brain tumor detection. This research utilizes convolutional neural networks, similar to our approach for detecting tomato leaf illness, highlighting the adaptability of deep learning frameworks across different fields to boost detection accuracy and processing efficiency. However, the study also identifies a major limitation: the high computational demands of these advanced models, which may be impractical in resource-constrained environments typical of agricultural settings. This highlights the necessity for developing models that balance high performance with efficient resource use in real-world applications.

In their study^22^, researchers classified brain tumors using advanced algorithms. Their research demonstrated that the VGG16 deep learning model outperformed traditional support vector machine (SVM) classifiers, achieving an impressive 97.86% accuracy with a five-layer convolutional neural network (CNN). However, they noted that such high accuracy might not be achievable with different or less balanced datasets. Additionally, the substantial computational requirements of deep learning models like VGG16 present practical challenges in settings with limited computing resources. These considerations are crucial when applying similar technologies to other fields, such as disease detection in agriculture, where resource limitations and diverse data are common obstacles.

In their paper^23^, the researchers explore with employment of machines-learning and deepL for MRI-based brain tumor diagnosis. They detail how these automated systems improve diagnostic speed, reliability, cost-effectiveness, and accuracy. Despite the advancements, the study notes significant challenges such as the dependency on high-quality data and the risk of overfitting, which could affect the models' applicability to broader scenarios. These insights are particularly relevant to similar technologies used in agricultural disease detection, highlighting both the potential and the limitations of applying deep learning methods across different scientific fields.

In this research^24^, a survey on computer vision applications in plant pathology is conducted, focusing on smart agriculture enhancements. They examined studies utilizing digital image processing and soft computing to boost crop productivity and quality through automated monitoring systems. While acknowledging the efficacy of computer vision in disease identification and classification, they noted challenges like the necessity for high-quality image data and difficulties in diverse agricultural settings. These insights inform our research into novel diagnostic tools for plant health.

## Experiment and procedure used

**Fig. 1:**
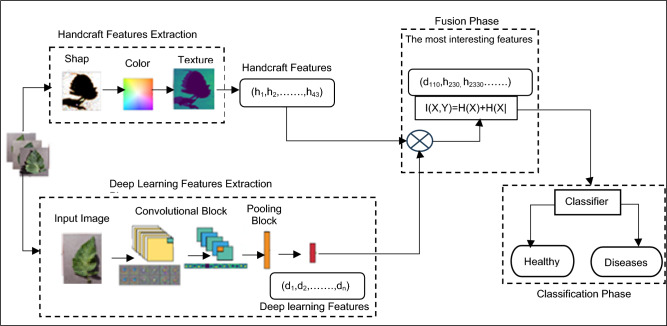
Proposed system blocks structure.

In the detailed presentation of our proposed methodology, as illustrated in Fig. 1 through a conceptual block diagram, we begin our exploration into the complexities of precision. The process starts with the crucial step of segmenting tomato leaf lesion images. This task is essential for distinguishing lesions from the surrounding healthy leaf tissue, despite challenges posed by overlapping structures such as veils and filaments^25^.

This segmentation achievement, notable in its own right, involves a series of steps including color space transformation and the application of mean threshold values to meticulously excerpt for Region of-Interest since digital imageries^26^.In process reveals core for-lesion, preparing it for further analysis. Once segmentation is complete, our focus shifts to the derived ROI, where we apply handcrafted features involving shape, color, and texture, each elaborately crafted through additional processing steps^27^. This detailed analysis provides valuable insights into the lesion’s characteristics.

However, our journey continues as we delve into deep learning. The foundational elements of our approach are derived from a specifically chosen Convolutional Neural Network (CNN) architecture. These features, informed by pre-trained models from ImageNet, enrich our analysis with advanced visual recognition capabilities^28^. Yet, our exploration does not end here; it reaches its climax in the process of feature fusion. Here, the Mutual Information (MI) criterion plays a crucial role in the seamless integration of all extracted features into a comprehensive vector, creating a unified analysis framework.

Empowered by this comprehensive feature vector, we advance to the final stage-using a classifier that has been refined with images of both benign and malignant tomato leaf lesions, sourced from the Plants Health’s Open-Access Images-Library-dataset. This classifier, equipped to make informed judgments, determines the condition of each unobserved tomato leaf lesion, identifying it as healthy or diseased.

### Data-preprocessing

In the expansive domain of image analysis, our investigation navigates through the coordinates (x, y), unveiling a narrative embedded within an initial image that holds the potential for significant revelations. This narrative reveals the presence of lesions accompanied by intriguing artifacts like curls, spots, and stains. To illuminate and extract the genuine essence of this story, we utilize the artistry of preprocessing, drawing from the insights of previous studies^8,15,19^. Specifically focusing on artifacts, our attention centers on the Gaussian filter—a tool meticulously crafted to enhance and obscure simultaneously. Its objective is precise: selectively targeting artifacts to delicately blur disorderly elements while safeguarding the geometric integrity of the lesion.

This Gaussian filter, a virtuoso of subtlety, is defined by the following incantation:

1 $$G\left(x,y\right)=\frac{1}{2\pi {\sigma }^{2}}\text{exp}\left(-\frac{{x}^{2}+{y}^{2}}{2{\sigma }^{2}}\right)$$

The modulation of this intricate interplay between blurring and preservation is governed by the variance ${\sigma }^{2}$ of the spatial kernel. In this scientific exploration, we elucidate the core of image refinement, where preprocessing tools and the Gaussian filter are instrumental techniques for enhancement, each application unveiling hidden truths.

Our exploration of the CIEL*a*b* color space reveals a nuanced framework that accurately mirrors human perception. Composed of three channels—L for lightness, a* for magenta to green, and b* for blue to yellow—this transformation, inspired by a sub-variant of the ABCD algorithm, does more than enhance aesthetics. It orchestrates an intricate dance to prevent channel correlations while preserving the perceptual essence of the image. The color space effectively captures even the darker hues of pigmented tomato leaf lesions compared to healthy leaves.

**Fig. 2:**
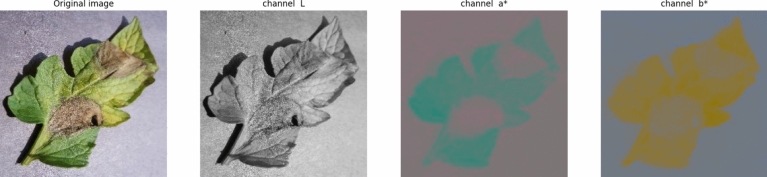
Visualization of original image and CIELab* channels (**a**) original image, (**b**) L* channel, (**c**) a* channel, (**d**) b* channel.

In color analysis, we employ mean thresholding, a digital chiaroscuro technique applied to I_L_, I_a_, and I_b_ channels shown in Fig. 2. This computational process unveils mean values, revealing subtle nuances and contrasts within the image, thus demonstrating the precision of image analysis.

2 $$\overline{{I }_{L}}=\frac{1}{mn}\sum_{x=1}^{m} \sum_{y=1}^{n} {I}_{L}(x,y)$$

3 $$\overline{{I }_{a}}=\frac{1}{mn}\sum_{x=1}^{m} \sum_{y=1}^{n} {I}_{a}\left(x,y\right),$$

4 $$\overline{{I }_{b}}=\frac{1}{mn}\sum_{x=1}^{m} \sum_{y=1}^{n} {I}_{b}\left(x,y\right),$$

In the intricate process of image manipulation, involving dimensions denoted as m and n, and spatial coordinates (x, y), luminance and chroma, represented by $\overline{{I }_{L}}$ , $\overline{{I }_{a}}$, and $\overline{{I }_{b}}$ channels in the CIELab* domain, undergo a transformation through thresholding. This computational operation reveals thresholded channel images that incorporate shadows and highlights, intricately interwoven to convey nuanced perceptions.

5 $${I}_{T{h}_{L}}\left(x,y\right)=\left\{\begin{array}{c}1,\hspace{0.25em}\hspace{0.25em}\hspace{0.25em}\hspace{0.25em}{I}_{L}(x,y)\ge \overline{{I}_{L}}\\ 0,\hspace{0.25em}\hspace{0.25em}\hspace{0.25em}\hspace{0.25em} \, {\text{o}}{\text{t}}{\text{h}}{\text{e}}{\text{r}}{\text{w}}{\text{i}}{\text{s}}{\text{e}} \, \end{array}\right.$$

6 $${I}_{T{h}_{a}}\left(x,y\right)=\left\{\begin{array}{c}1,\hspace{0.25em}\hspace{0.25em}\hspace{0.25em}\hspace{0.25em}{I}_{a}(x,y)\ge \overline{{I}_{a}}\\ 0,\hspace{0.25em}\hspace{0.25em}\hspace{0.25em}\hspace{0.25em} \, {\text{o}}{\text{t}}{\text{h}}{\text{e}}{\text{r}}{\text{w}}{\text{i}}{\text{s}}{\text{e}} \, \end{array}\right.$$

7 $${I}_{T{h}_{b}}\left(x,y\right)=\left\{\begin{array}{c}1,\hspace{0.25em}\hspace{0.25em}\hspace{0.25em}\hspace{0.25em}{I}_{b}(x,y)\ge \overline{{I}_{b}}\\ 0,\hspace{0.25em}\hspace{0.25em}\hspace{0.25em}\hspace{0.25em} \, {\text{o}}{\text{t}}{\text{h}}{\text{e}}{\text{r}}{\text{w}}{\text{i}}{\text{s}}{\text{e}} \, \end{array}\right.$$

**Fig. 3:**
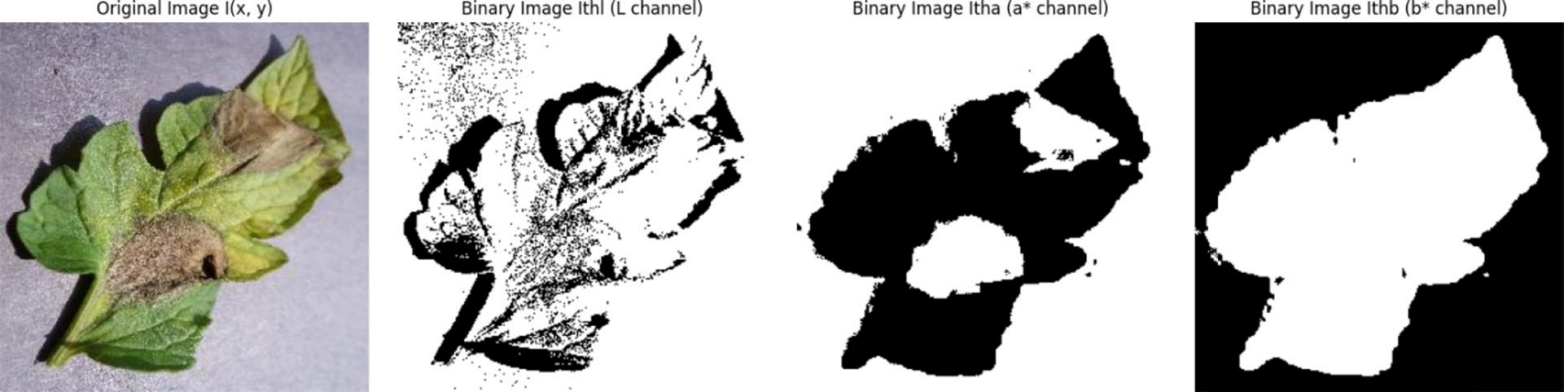
Results of the thresholding stage in CIELab color space. original image-I(x, y), binary-image Ithl (L channel), binary image Itha (a* channel), binary image Ithb (b* channel).

In the process of image manipulation, binarized images, namely ${I}_{T{h}_{L}}$, ${I}_{T{h}_{a}}$, and ${I}_{T{h}_{b}}$ shown in Fig. 3, undergo a coordinated binary conjunction, resulting in the creation of a binary mask image, ${I}_{bin}$ .

This mask, derived from the original image, serves as an illuminating guide for subsequent analysis.

8 $${I}_{bin}\left(x,y\right)={I}_{T{h}_{L}}\cap {I}_{T{h}_{a}}\cap {I}_{T{h}_{b}}$$

The intricate process involves transforming the mathematical model ${I}_{bin}$ , using a powerful 5 × 5-kernel median filter to eliminate persistent artefacts. The bounding box algorithm takes center stage, creating a geometrically intricate space for the sought-after object, demarcated by cryptic coordinates. The resulting Region of Interest (ROI), ${I}_{o}$, becomes a treasure trove of insights in computer vision, paving the way for further computations and classification model refinement within the embrace of mathematical obscurity.

### Handcraft features

In the realm of image analysis, handcrafted features often originate from visual indicators that encompass aspects like color, texture, shape, and various perceptual attributes. These meticulously chosen features are designed with precision to encapsulate pertinent information about the visual composition of an image.

#### Shape features

In the domain of image analysis, shape features encompass quantifiable metrics or descriptors used to delineate the geometric attributes of entities present in an image. These features convey details about the configuration, composition, and spatial organization of discernible elements within the visual content. To describe how humans perceive an object's form, shape, or geometric aspects, specific shape features are utilized^29^.

Common shape features include:

- Area: the measure of the extent of the object's surface within its boundaries
  9 $$\text{Area }=\sum_{x=1}^{m} \sum_{y=1}^{n} {I}_{bin}\left(x,y\right)$$
- Perimeter: the length of the boundary outlining the object.
  10 $$\text{Perimeter }=\sum_{i=1}^{m} \sqrt[2]{\left({x}_{i}-{x}_{i-1}\right)+\left({y}_{i}-{y}_{i-1}\right)}$$
- Circularity: indicates how closely the shape resembles a perfect circle and is computed as a function of the shape's perimeter and area.
  11 $$\text{Circularity }=\frac{4\pi \cdot {\text{ Area }}^{2}}{{\text{ Perimeter }}^{2}}$$
- Diameter: offering quantitative metrics delineating the dimensions and spatial attributes of entities within the digital canvas.
  12 $$\text{Diameter }=\sqrt[2]{\frac{1}{2}\left({\mu }_{\text{2,0}}+{\mu }_{\text{0,2}}\right)\pm \sqrt[2]{4{\mu }_{\text{1,1}}^{2}-{\left({\mu }_{\text{2,0}}-{\mu }_{\text{0,2}}\right)}^{2}}}$$
  ${\mu }_{\text{2,0}}$, ${\mu }_{\text{0,2}}$ and ${\mu }_{\text{1,1}}$ denote with central moments characterizing spatial distribution of the object.
  - ${\mu }_{\text{2,0}}$ is the second-central moment about the x-axis,
  - ${\mu }_{\text{0,2}}$ is the second central minute about the y-axis,
  - ${\mu }_{\text{1,1}}$ is the central-moment about the x–y plane.

- Eccentricity: describes how elongated or stretched a shape is.
  13 $$\text{Eccentricity }=\frac{{\left({\mu }_{\text{0,2}}-{\mu }_{\text{2,0}}\right)}^{2}+4{\mu }_{\text{1,1}}}{A}$$

#### Colour features

Descriptors related to the color distribution in an image, including histograms or statistics of color channels, play a significant role. In the agricultural exploration of Tomato Leaf Diseases (TLD), colors, guided by the ABCD rule, assume a pivotal role. Statistical traits derived from color spaces serve as essential tools

14 $${\text{Min}}_{ch}=min\left[{I}_{ch}(x,y)\right]$$

15 $${\text{Max}}_{ch}=max\left[{I}_{ch}(x,y)\right]$$

16 $${\text{Var}}_{ch}=\text{var}\left[{I}_{ch}\left(x,y\right)\right]$$

17 $${\text{Mean }}_{ch}=\overline{\left[{I}_{ch}(x,y)\right]}$$

This image of a selected channel for the Tomato-Leaf Disease (TLD) image is represented by *I*_*ch*_(*x, y*) in the R–G–B/CIEL*a*b* colour spaces.

#### Textures-features

In the domain of image analysis, the Grey Level Co-occurrence Matrix (GLCM)^30^, reveals statistical textural properties in tomato leaf disease (TLD) portraits. The GLCM's normalized element ${P}_{d}(i,j)$, depicts the frequency of a grey level at a specific pixel locale, guided by ${N}_{g}$, ${\sigma }_{x}$ and ${\sigma }_{y}$ , ${\mu }_{x}$ and ${\mu }_{y}$ vigilantly maintain the harmony of grey level patterns in TLD photos through their roles in means and standard deviations.

18 $${\mu }_{x}=\sum_{i=1}^{{N}_{g}} \sum_{j=1}^{{N}_{g}} i{P}_{d}\left(i,j\right)$$

19 $${\mu }_{y}=\sum_{i=1}^{{N}_{g}} \sum_{j=1}^{{N}_{g}} j{P}_{d}\left(i,j\right)$$

20 $${\sigma }_{x}=\sqrt{\sum_{i=1}^{{N}_{g}} \sum_{j=1}^{{N}_{g}} {\left(i-{\mu }_{x}\right)}^{2}{P}_{d}(i,j)}$$

21 $${\sigma }_{y}=\sqrt{\sum_{i=1}^{{N}_{g}} \sum_{j=1}^{{N}_{g}} {\left(j-{\mu }_{y}\right)}^{2}{P}_{d}(i,j)}$$

In this-study, we utilize 13 texture features as follows:

22 $$ASM=\sum_{i=1}^{{N}_{g}} \sum_{j=1}^{{N}_{g}} {P}_{d}^{2}\left(i,j\right)$$

23 $$\text{Contrast }=\sum_{i=1}^{{N}_{g}} \sum_{j=1}^{{N}_{g}} (i-j{)}^{2}{P}_{d}\left(i,j\right)$$

24 $$\text{Correlation }=\sum_{i=1}^{{N}_{g}} \sum_{j=1}^{{N}_{g}} {P}_{d}\left(i,j\right)\frac{\left(i-{\mu }_{x}\right)\left(j-{\mu }_{y}\right)}{{\sigma }_{x}{\sigma }_{y}}$$

25 $$\text{Variance }=\sum_{i=1}^{{N}_{g}} \sum_{j=1}^{{N}_{g}} (i-\mu {)}^{2}{P}_{d}\left(i,j\right)$$

26 $$IDM=\sum_{i=1}^{{N}_{g}} \sum_{j=1}^{{N}_{g}} \frac{1}{1+(i-j{)}^{2}}{P}_{d}\left(i,j\right)$$

27 $$\text{Entropy }=-\sum_{i=1}^{{N}_{g}} \sum_{j=1}^{{N}_{g}} {P}_{d}\left(i,j\right)\cdot \text{ln}\left[{P}_{d}\left(i,j\right)\right]$$

Additionally, we use texture features based on different statistics, utilizing the probability ${P}_{x-y}\left(k\right)$:

28 $${P}_{x-y}\left(k\right)=\sum_{i=1}^{{N}_{g}} \sum_{j=1}^{{N}_{g}} {P}_{d}\left(i,j\right),k=\text{0,1},\dots ,{N}_{g}-1$$

These features include:

29 $$\text{Sum Variance }=\sum_{k=2}^{2{N}_{g}} {\left(k-{\mu }_{x+y}\right)}^{2}{P}_{x+y}\left(k\right)$$

30 $$\text{Sum Entropy }=-\sum_{k=2}^{2{N}_{g}} {P}_{x+y}\left(k\right)\text{log}\left[{P}_{x+y}\left(k\right)\right] $$

31 $$\text{Difference Variance }=\sum_{k=0}^{{N}_{G}-1} {\left(k-{\mu }_{x-y}\right)}^{2}{P}_{x-y}\left(k\right)$$

32 $$\text{Difference Entropy }=-\sum_{k=0}^{{N}_{g}-1} {P}_{x-y}\left(k\right)\text{log}\left[{P}_{x-y}\left(k\right)\right]$$

33 $$IM{\text{Corr}}_{1}=\frac{H(XY)-H\left(X{Y}_{1}\right)}{max[H(X)H(Y)]}$$

34 $$IM{\text{Corr}}_{2}=\sqrt{1-\text{exp}\left\{-2\left[H\left(X{Y}_{2}\right)-H(XY)\right]\right\}}$$

We can define *H(X), H(Y), H(XY), H(XY1), and H(XY2*) by way of follow:

35 $$H\left(X\right)=-\sum_{i=1}^{{N}_{g}} {P}_{x}\left(i\right)\cdot \text{log}\left[{P}_{x}\left(i\right)\right]$$

36 $$H\left(Y\right)=-\sum_{i=1}^{{N}_{g}} {P}_{y}\left(i\right)\cdot \text{log}\left[{P}_{y}\left(i\right)\right]$$

37 $$H\left(XY\right)=-\sum_{i=1}^{{N}_{g}} {P}_{d}\left(i,j\right)\cdot \text{log}\left[{P}_{d}\left(i,j\right)\right]$$

38 $$H\left(XY1\right)=-\sum_{i=1}^{{N}_{g}} \sum_{j=1}^{{N}_{g}} {P}_{d}\left(i,j\right)\cdot \text{log}\left[{P}_{x}\left(i\right)\cdot {P}_{y}\left(j\right)\right]$$

39 $$H\left(XY2\right)=-\sum_{i=1}^{{N}_{g}} \sum_{j=1}^{{N}_{g}} {P}_{x}\left(i\right)\cdot {P}_{y}\left(j\right)\cdot \text{log}\left[{P}_{x}\left(i\right)\cdot {P}_{y}\left(j\right)\right]$$

### Deep learning features

Deep learning features in image analysis refer to the high-level representations automatically learned by deep neural networks (DNNs) during training. These features capture complex patterns and hierarchical relationships in the data, allowing the network to discern intricate details and make predictions. In the context of tomato leaf-disease-classification, deep learning variables stay put in since layers within CNN-architectures, leveraging the network's ability to automatically learn relevant representations from raw input data^31^. These features contribute to the overall understanding of image content, aiding in the identification and classification of tomato Leaf diseases. Utilizing discrete convolution operations, represented by

40 $$W\left(i,j\right)=\left(K*I\right)\left(i,j\right)=\sum_{m} \sum_{n} I\left(i-m,j-n\right)K\left(m,n\right) $$

41 $$y=z\left(W*X\right)+b$$

This neural network operation reveals symbolic elements like X, z, and W, representing input, activation function, and computed values within filters, respectively, with the addition of bias denoted by b. Designing an optimal CNN becomes a journey through the complex dimensions of filter depth and size, posing challenges that necessitate exploration in the nuanced realms of neural design to uncover effective configurations within the detailed framework.

#### Transfers-learning

In intricate landscape of deep learning-where knowledge requires abundant data, a formidable challenge unfolds. This undertaking is fraught with complexity due to the substantial demands for ample data in the digital realm. To address this challenge, we employ the time-honored technique of transfer learning, a venerable method drawn from extensive research^32^.

Learned with transfer techniques for the practice to transferring information as of basis errands to the framework of a board task, reflects the essence of human learning. Our journey begins with foundational elements: colors, shapes, textures—the basic facets of perception that precede the emergence of more advanced knowledge, such as distinguishing a chair from an apple. In the formal terminology of this practice, we navigate the realms of symbols and domains^29^. Within this context D, symbolizing wisdom, encompasses two cryptic facets

42 $$D=\left\{\chi ,P\left(X\right)\right\}$$

where $\chi$ stands for feature vectors inside of feature-space c, too P(X) is a marginal-distributions. Given a task T that has two parts:

43 $$T=\left\{\gamma ,P(Y|X)\right\}=\left\{\gamma ,\eta \right\};Y=\left\{{y}_{1},\dots ,{y}_{n}\right\},{y}_{i}\in \gamma $$

#### Features extractor

The convolutional neural network serves equally a proficient conductor in extracting cryptic features from the image I(x, y) within the domain D_T_, aligning with the broader concept of feature extraction. Feature extraction, a crucial element in machine learning and computer vision, involves the automatic identification and extraction of essential patterns from raw data, with CNNs acting as potent feature extractors. This process is vital for reducing data dimensionality while retaining crucial information, particularly in tasks like image recognition, where identifying pertinent features is crucial for accurate analysis.

44 $$W=\left\{{w}_{1},\dots ,{w}_{n}\right\};{w}_{1}\in {\mathbb{R}}^{M\times N\times h}$$

Here, M, N, and h stay stated dimensions of neural-network architecture, while W stands for the weights computed by the feature extractor. This process results in:

45 $$P=W\left(I\left(x,y\right)\right)=\left\{{w}_{1}\left(I\left(x,y\right)\right),\dots ,{w}_{n}\left(I\left(x,y\right)\right)\right\};\in {\mathbb{R}}^{M\times N\times h}$$

Pooling transformations are then applied:

46 $$Q=f\left(0,P\right)=\left\{f\left(0,{w}_{1}\left(I\left(x,y\right)\right)\right),\dots ,f\left(0,{w}_{n}\left(I\left(x,y\right)\right)\right)\right\}$$

wherever f(.) denotes a mapping function.

#### Deep learning-architectures

In computational advancement, a diverse array of architectural configurations is strategically employed, meticulously chosen for their distinguished lineage—a lineage marked by a history of remarkable precision and minimal errors. These revered achievements are cultivated during the esteemed odyssey termed the ImageNet task^31^. Observe the enigmatic assembly:

- VGG-16 and VGG-19, acknowledged as ancient sages^33^.
- MobileNet, a digital wanderer and nomad of algorithms^34^.
- ResNet-50, a fortress symbolizing resilience and strength, shaped in the crucible of neural battles^30^.
- Inception v3, a visionary dream weaver of dimensions and depths^32^.
- Xception, a cipher unraveling the enigma of complexity^32^.
- DenseNet-201, embodying density where pixels converge into a cosmic singularity^35^.

In our scholarly exploration, these eminent architectural achievements, regarded with deep respect, have reached the pinnacle of excellence in image classification and feature extraction. For creations outcomes off as singular featured vectors, a manifestation of meticulous cohesion that blends the artistry of handcrafted features with the profound insights derived from each selected architecture—an illustrative demonstration of the fusion between pixels and patterns

47 $$F={D}_{\text{eep }}\left[\#\text{ Features Extracted }\right]\cup {H}_{\text{and }}\left[\text{ Asymetry, Area },..,\text{ Entropy, Contrast },\text{ Std..., Max, Min },..\right]$$

With comprehensive structure our digital framework, we delve into the essence of feature extraction, where an abundance of features—unveiled by the enigmatic CNN architecture—converges and intertwines with 43 meticulously designed features. This amalgamation, akin to a precise choreography of data points, signifies the emergence of vector F, a monumental reservoir of information. Within this vector, the distinction between neural processing and human craftsmanship dissipates, forming an intricately woven tapestry of insights.

### Overview of the algorithm

In the realm of our proposed Computer-Aided Diagnosis (CAD) system, we undertake an algorithmic exploration to extract intrinsic characteristics from Tomato Leaf images. This intricate process comprises four stages: Preprocessing, handcrafted features crafting, deep learning feature orchestration, integration of these revelations.

#### Foundational phase

In the foundational phase, we establish the groundwork as follows:

1. Initiation begins with 'Image' invocation, the entry point to our digital domain.
2. The Gaussian Filter, governed by Eq. (1), imposes transformations upon our vision.
3. The metamorphosis from CIELab* to RGB color transformation unveils intrinsic colors.
4. In the CIELab* realm, the convergence of Image Channels, IL, Ia, and Ib, occurs in the sacred ILab.
5. Mean values of IL, Ia, and Ib emerge through Eqs. (2) to (4), resembling celestial harmonies with a mysterious purpose.
6. Mean thresholding, a digital procedure per channel, is invoked to generate binary masks, summoning the essence of pixels.
7. Equation (8) materializes the Intersection Binary image Ibin, a revelation of the pixelated cosmos.
8. The Median Filter, with 5 × 5 configuration, purifies the image.
9. The Bounding Box Algorithm gracefully computes coordinates for the elusive Region of Interest (ROI), akin to celestial coordinates.
10. The prophecy of coordinates is fulfilled as I and ILab collaborate to create the ROI image, their destinies intertwined in the digital manipulation of pixels.

#### Handcrafted feature extraction phase

Navigating the empirical realm of handcrafted feature extraction, the unfolding acts resonate as procedural components in a technical manuscript:

11. The procedure begins with the 'Iroi Image,' a data structure representing the essence of our ROI.
12. Equations (9)–(13) articulate the computation of shape characteristics—area, perimeter, circularity, diameter, and eccentricity. In parallel, the calculation of asymmetry emerges, defined by Eqs. (9)–(11).
13. Color Features manifest from the soul of Iroi through Eqs. (14) to (17), a quantitative representation of hues and shades guided by mathematical principles.
14. Equations (18)–(40) unveil texture features as numerical representations, a testament to the pixelated information concealed within Iroi.
15. Features amalgamate as quantitative values to form 'H,' a numerical ensemble of empirical measures, a product of our systematic methodology.
16. 'H' materializes as a numeric entity, a result embedded in the digital framework, a product of our empirical methodology.

#### Deep learning features extraction phase

Embarking on the empirical realm of deep learning features, the ensuing procedures meticulously construct the framework of understanding:

17. 'ROI Image Iroi' is introduced as a data structure representing the gateway to the neural cosmos.
18. Preordained 'Wi' weights, imparted by the selected CNN architecture, stand ready for application.
19. 'Wi' integrates with 'Iroi,' resulting in a fusion of neural potential.
20. Deep learning capabilities, 'D,' emerge as computational outcomes, bearing the quantitative representation of pixel and pattern knowledge.
21. Deep Learning Features arise from the depths of 'D,' a numerical composition of empirical insight awaiting integration into the comprehensive knowledge structure.

#### Feature fusion phase

As we approach the culmination of synthesis, the final procedures unfold:

22. 'D' and 'H' assume their roles as custodians of knowledge, positioned at the brink of enlightenment.
23. The fusion of 'H' and 'D' commences, analogous to the integration of celestial bodies, revealing the ultimate artifact—the comprehensive ensemble of extracted features, 'F.'
24. From the amalgamation process, the Full set of extracted features, 'F,' emerges as a numeric revelation awaiting integration into the digital repository of empirical knowledge.

### Feature selection

In the expansive arena of data intricacies, where features abound, the challenge arises in navigating high-dimensional dimensions. Feature Selection emerges as a crucial solution, utilizing statistical tools like chi-squared ( ${\chi }^{2}$), ANOVA, and linear discriminant analysis (LDA)^36^. The dataset is depicted as a high-dimensional matrix, a mosaic of knowledge ready for refinement a transformation to unveil concealed wisdom within the digital realm.

48 $$\mathbf{X}\in {\mathbb{R}}^{n\times p}$$

In this vast landscape, 'n' represents instances, and 'p' signifies features for each element. The matrix 'X' becomes the vessel of extracted knowledge. The quest is to sculpt this matrix, distilling the cacophony of data into a meaningful subset. The mission: to select relevant features marked by the classification label 'y.' In this expansive setting, the objective is to prune the data, revealing the essence within digital realms, distilling wisdom into a select few.

49 $${\mathbf{X}}_{S}\in {\mathbb{R}}^{n\times k}$$

In the evolving narrative, the emergence of reduced data as ${\mathbf{X}}_{S}$ undergoes a transformative process, mirroring the essence of 'n.' As the dance unfolds, 'k' materializes—an enigmatic subset of features selected with discernment, embodying wisdom as k <  < p.

### Mutual information

In the realm of data exploration, our focus shifts to Mutual Information (MI), a key principle in navigating complexity. Within the digital landscape, we embrace MI as our metric a guiding compass through intricacies. MI, fundamentally, encapsulates the interplay of entropy, illustrating the dynamic relationship between information and uncertainty. This mathematical expression engraves the essence of MI into the records of our study.

50 $$I\left(X,Y\right)=H\left(X\right)-H(X|Y)=\sum_{y\in Y} \sum_{x\in X} P\left(X\cap Y\right){\text{log}}_{2}\left(\frac{P\left(X\cap Y\right)}{P\left(X\right)P\left(Y\right)}\right)$$

In the intricate realm of multivariate realities, where Y unfolds as the ensemble y_1_,…, y_n_, and X, a realm of possibilities, manifests as x_1_,…, x_n_, we delve into the cryptic embrace of entropy. H(X), portraying uncertainty, becomes the essence of randomness the pulse of a random variable, a digital oracle resonating with the mysteries of the unknown. Simultaneously, H(X|Y), the conditional entropy, intertwines two elusive entities in a complex interplay an unfolding exploration through the threads of probability and knowledge.

51 $$H\left(x\right)=-\sum_{i=1}^{n} P\left({x}_{i}\right){\text{log}}_{2}P\left({x}_{i}\right)$$

As we delve into Mutual Information (MI), a process unfolds transforming continuous data into discrete echoes within bins. Witness the binning approach, shaping data into bins like constellations in the digital realm. Here, MI reveals itself an approximation derived from the essence of bins, on the edge between the tangible and the ephemeral.

52 $$I\left(X,Y\right)={\langle \text{log}\frac{p\left({x}_{i},{b}_{i}\right)}{p\left({x}_{i}\right)p\left({b}_{i}\right)}\rangle }_{i}$$

In the aftermath of applying the MI method, a new vector emerges with MI values, each feature telling its story within the realm of knowledge. A threshold, derived from the mean MI value, acts as a central reference point, determining which features are retained.

Features below the threshold are discarded, while those above find refuge. This MI-based approach ensures features earn their place based on informativeness, departing from conventional methods. Information measures reign supreme, justifying the value of each feature in the sacred act of classification.

Tables 1 and 2 unveil features, some with low MI values and others elevated. These tables serve as canvases, illustrating the process of selection and influence a classification problem played out in the subtle nuances of data. Table 1 provides examples of features with low Mutual Information values, while Table 2 showcases features with high Mutual Information values.

**Table 1:** Example of features with low mutual information values.

Feature	Mutual info. value
Feature_1	2.135e−7
Feature_2	1.798e−5
Feature_3	2.323e−5
Feature_4	2.486e−5
Feature_5	3.012e−5
Feature_6	3.155e−5
Feature_7	3.657e−5
Feature_8	4.132e−5
Feature_9	5.278e−5
Feature_10	5.391e−5
……	……

**Table 2:** Example of features with high mutual information values.

Feature	Mutual info. value
Feature_A	0.10648234981570472
Feature_B	0.09871263827194205
Feature_C	0.07892437510621893
Feature_D	0.07584628543053912
Feature_E	0.07126492653472183
Feature_F	0.06591046385764922
Feature_G	0.06481375421034287
Feature_H	0.06197825549238671
Feature_I	0.05977163290120354
Feature_J	0.05769281329476201
……	……

### Interpretability analysis

#### Dual-branch design benefits

The dual-branch design in our proposed methodology offers several benefits:

- Enhanced feature representation: by incorporating multiple branches, each focusing on different aspects or modalities of the input data, we can capture richer and more diverse feature representations. This can lead to improved classification performance by allowing the model to learn from complementary information.
- Robustness to variations: different branches may specialize in capturing features relevant to specific variations or characteristics within the data. For example, one branch may focus on texture features while another on color features. This can enhance the model's robustness near differences cutting-edge inputs data, such as variations now illumination conditions before disease manifestations.
- Interpretability and explain-ability: the dual-branch architecture can facilitate interpretability and explain ability to model estimates. By analyzing and contributions in each branch to final decision, we can gain insights into which features or modalities are most influential in the classification process.

#### Integration of heat maps

Providing heat maps of tomato leaf samples to illustrate the attention regions of the CNN, we propose the following:

- Visualization technique: we will integrate heat map visualization methods likes Grad-CAM (Gradients Weighted Classes-Activations-Mappings) or else CAM (Class-Activations-Mappings), into our methodology.
- Illustrating attention regions: these heat maps will highlight the regions of the tomato leaf images that the CNN model focuses on when making classification decisions. By visualizing the at-attention regions, we can provide insights into which parts of the images are most informative for disease classification.
- Interpretation and analysis: heat maps will not only enhance the interpretability of our model but also enable researchers and stakeholders to analyze and validate the model's decision-making process. This can lead to a better understanding of the underlying mechanisms driving the classification outcomes.

## Results and discussion

### Data set

**Fig. 4:**
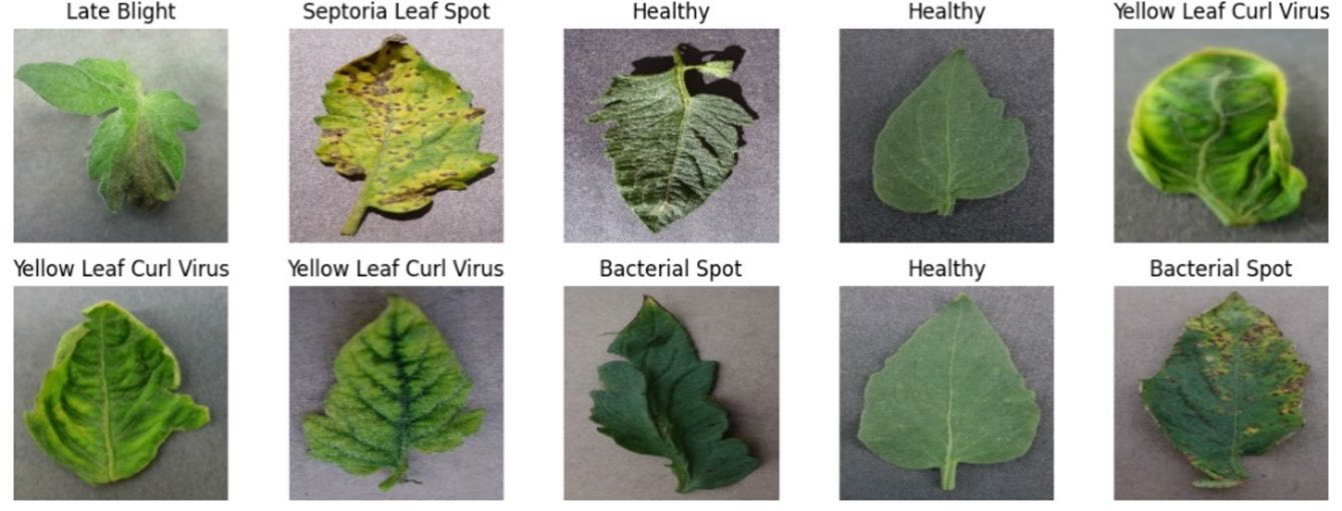
A sample images of different phenotypes of tomato plants.

In the realm of botanical knowledge, Hughes & Salathé's Plant Health Open Access Image Library stands as a valu-able source, providing us with a sacred dataset featuring various tomato 1eaf diseases^16^. As we delve into the details presented in Table 3 and Fig. 4, we encounter a digital representation that unfolds, telling stories of both thriving, healthy tomato crops and the diverse ailments that challenge them—a digital repository that serves as a testament to the ongoing battle of plants against afflictions.

**Table 3:** Data-set statistics.

Labels	Categories	Samples size
1	Bacterial spot	900 samples
2	Early blight	500 samples
3	Healthy	701 samples
4	Late blight	800 samples
5	Leaf mold	400 samples
6	Septoria leaf spot	800 samples
7	Spider mites	800 samples
8	Target spot	700 samples
9	Mosaic virus	160 samples
10	Yellow leaf curl virus	1540 samples

Images capturing samples of both healthy tomato crops and nine distinct diseases were collected from the Plant Village dataset. These images underwent segmentation, where background pixels in all three channels were set to 0, except those related to a leaf. A comprehensive dataset consisting of 7,311 samples from 9 categories of both healthy and diseased samples was utilized. Employing a selected deep-learning method, the initial 256X256 pixel input images were reduced to 224 X 224 pixels. The dataset was partitioned into a training set (75%) and a test set (25%). Extracted features underwent Z-score normalization processing

53 $$Z=\frac{x-\mu }{\sigma }$$

X represents the feature value, $\mu$ represents the mean, and $\sigma$ is the standards-deviations.

### Balanced data

In the intricate realm of data transformation, the ISIC dataset unfolds a narrative of imbalanced proportions. SMOTE, an augmentation technique, endeavors to restore equilibrium by generating new data points through K-NN clustering and Euclidean distance. This iterative process persists until balance is achieved. Serving as a guardian against imbalance, SMOTE oversamples feature to create a balanced and fused dataset. Guided by MI, we incorporate SMOTE in this ritual. Introducing the 'Geometric Mean (Gmean),' a metric emerging from imbalance, seeks harmony—a digital symphony resolving the imbalances that persist

54 $${G}_{\text{mean }}=\sqrt[2]{\text{ Sensitivity }\cdot \text{ Specificity }} $$

To assess the classifier's performance, dominance measures the prevailing relationship between the majority and minority classes.

55 $$\text{Dominance }=\text{ Sensit}\text{ivity }-\text{ Specificity}$$

The Index of Balance-Accuracy (IBA) is an enactment metrics cutting-edge classifications designed toward enhance sensitivity in imbalanced domains

56 $$IBA=\left(1-\text{ Dominance }\right)\cdot {G}_{\text{mean }}^{2}$$

These metrics aim to preserve the significance of the majority class while slightly favouring classifiers models per a advanced measurement of accurately predicting the minority class.

### Performance evaluations of metrics

To assessed performance using standard performance indicators, which encompass F-score, accuracy, sensitivity, specificity, and precision:

57 $$\text{Accuracy }=\frac{tp+tn}{tp+tn+fp+fn}$$

Across the entire set of analyzed items, accuracy quantifies the correct classifications.

58 $$\text{Sensitivity }=\frac{tp}{tp+fn}$$

Sensitivity, often known as recall, measures the identification of positive attributes.

59 $$\text{Specificity }=\frac{tn}{tn+fp}$$

Specificity quantifies the accurate recognition of negative elements.

60 $$\text{Precision }=\frac{tp}{tp+fp}$$

Precision assesses the accurate identification of positive elements among all positive classifications.

61 $${F}_{\text{Score }}=\frac{2tp}{2tp+fp+fn}$$

### Results and discussion

In our experiments, the tables illustrate the classification performance of our system, highlighting the balance achieved in our data. Table 5 percent these performances in our system thru deep learning architectures besides customized features. The integration of handcrafted features using CNN architectures significantly improves the automate detect and classify of tomato leaf diseases, as shown trendy Table 4 compared to Table 5. Our results demonstrate consistent improvements across multiple metrics evaluations, containing accuracy’s, sensitivity, specificity, precision, F1-Scores, AUC-score, G-Means, and IBA.

**Table 4:** Tomato leaf CNN with handcrafted features results.

Architecture	Acc.Train	Acc.Test	Sensitivity	Specificity	Precision	F-score	AUC	G-mean	IBA
VGG-16	87.50	82.30	78.91	0.84	87.32	82.11	82.67	0.84	0.70
VGG19	89.20	86.11	81.87	0.86	89.32	85.24	85.91	0.86	0.75
Mobilenet v1	90.65	88.51	83.72	0.88	91.74	87.66	88.21	0.88	0.78
Mobilenet v2	91.90	88.84	85.13	0.89	91.28	88.43	88.91	0.89	0.79
ResNET-50	89.75	86.92	80.32	0.87	92.04	85.90	86.54	0.87	0.76
DenseNET-201	90.40	87.77	82.05	0.87	90.89	86.98	87.61	0.87	0.77
Inception V3	90.85	87.44	83.28	0.87	89.71	87.01	87.36	0.87	0.76
Xception	89.95	86.99	82.73	0.87	90.15	86.54	86.87	0.87	0.75

**Table 5:** Tomato leaf CNN without handcrafted features results.

Architecture	Acc.Train	Acc.Test	Sensitivity	Specificity	Precision	F-score	AUC	G-mean	IBA
VGG16	85.50	80.30	78.00	0.81	84.00	80.00	81.50	0.81	0.67
VGG19	87.20	83.11	79.87	0.83	87.32	82.24	83.91	0.83	0.71
Mobilenet v1	88.65	85.51	81.72	0.86	89.74	85.66	86.21	0.86	0.74
Mobilenet v2	89.90	86.84	82.13	0.87	90.28	86.43	87.91	0.87	0.76
ResNET-50	88.75	85.92	81.32	0.85	91.04	85.90	86.54	0.85	0.72
DenseNET-201	89.40	86.77	82.05	0.86	89.89	86.98	87.61	0.86	0.74
Inception V3	89.85	86.44	82.28	0.86	88.71	86.01	86.36	0.86	0.75
Xception	88.95	85.99	82.73	0.86	89.15	85.54	86.87	0.86	0.73

Specifically, training and testing accuracies were higher with the inclusion of handcrafted features. For instance, MobileNet v2’s training accuracy increased from 89.90 to 91.90%, and testing accuracy rose from 86.84 to 88.84%. Sensitivity and specificity also showed improvements, indicating a better true positive rate and true negative rate, respectively. Precision and F-Score, which measure the accuracy of positive predictions and the balance between precision and recall, respectively, were also enhanced.

Table 6 presents a comparative analysis of our proposed Computer-Aided Diagnose created happening in MobileNet v2 architecture, against previous CAD systems. The results indicate notable improvements in several key metrics. Our method addresses imbalanced data issues present in previous CAD systems by working with balanced data, resulting in more reliable diagnostics. Furthermore, our system uses fused data, which enhances the dataset's richness, and supports multiclass classification, unlike the binary classification approach used in earlier methods. This comprehensive performance comparison underscores the superiority and versatility given proposed CAD-system in identifying and categorising tomato leaf disease.

**Table 6:** Comparison between proposed CAD and previous CADs.

Metric	^16^	^30^	Proposed method (Mobilenet v2 architecture)
Accuracy	85.20	90.50	91.90
Sensitivity	78.10	84.10	85.13
Specificity	89.50	–	89.78
Precision	83.50	90.80	91.28
F-score	–	–	87.21
G-mean	–	–	0.89
IBA	–	–	0.80
Imbalanced-data	Yes	No	No
Fused-data	No	No	Yes
Classifications-types	–	Binary	Multi-classes

**Fig. 5:**
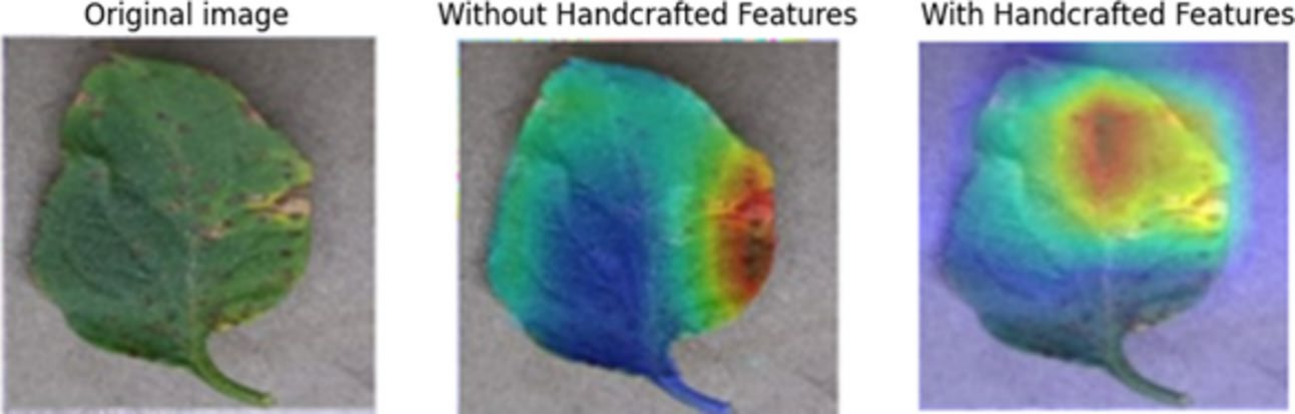
Grad-CAM visualizations for tomato leaf disease detection using original image, with and without handcrafted features.

In contrast, Fig. 5, which integrates handcrafted features, demonstrates a more focused heatmap on the diseased regions, indicating better localization accuracy. This suggests that the combination of handcrafted features and deep learning enhances the model's ability to accurately identify disease-affected areas. This comparison highlights the advantage of using a hybrid approach, improving the model's interpretability and performance in disease detection.

In summary, combining handcrafted features with CNNs significantly boosts performance in tomato leaf disease detection and classification, providing more reliable and accurate results. This approach effectively captures additional information that may be overlooked by CNNs alone, leading to comprehensive improvements across all evaluated metrics.

## Conclusion and future work

This research introduces an innovative computer-aided diagnosis-system meant precise detection and classification of tomato leaf diseases, merging traditional ABCD rule-based features with advanced deep learning methodologies. This integration surpasses conventional CAD frameworks by incorporating sophisticated data handling and analysis techniques. Utilizing transfer learning, the system bridges the gap between theoretical knowledge and practical application, which represents a significant shift from standard practices.

The implementation of the Mutual Information (MI) metric plays a pivotal role in enhancing the feature fusion process, ensuring optimal utilization of each feature for improved accuracy. Empirical results validate the system's efficacy, achieving an impressive 92% accuracy rate with an Index of Balanced Accuracy (IBA) of 0.80. These metrics affirm the system's capability to serve as a robust diagnostic tool in the agricultural sector.

Future directions for this research include expanding its capabilities to encompass multiclass classification, which will allow for broader applications in diagnosing a variety of plant diseases. This advancement aims to refine the integration of feature sets to boost diagnostic precision across diverse agricultural challenges.

## Data Availability

The datasets used during the current study are available in Kaggle at https://www.kaggle.com/datasets/cmgonadev/tomatos, specifically the universal Plant Village dataset, which can be accessed at Plant Village Tomato Leaf Dataset.
